# Alterations in the topological organization of the default-mode network in Tourette syndrome

**DOI:** 10.1186/s12883-023-03421-1

**Published:** 2023-10-30

**Authors:** Yue Yang, Hua Yang, Chunmei Yu, Fang Ni, Tao Yu, Rong Luo

**Affiliations:** 1 Department of Pediatrics, West China Second University Hospital, Sichuan University, Chengdu, 610041 China; 2 Key Laboratory of Obstetric & Gynecologic and Pediatric Diseases and Birth Defects of Ministry of Education, Sichuan University, Chengdu, 610041 China

**Keywords:** Tourette Syndrome, EEG, Default-mode network, Topological properties

## Abstract

**Background:**

The exact pathophysiology of TS is still elusive. Previous studies have identified default mode networks (DMN) abnormalities in patients with TS. However, these literatures investigated the neural activity during the tic suppression, not a true resting-state. Therefore, this study aimed to reveal the neural mechanism of Tourette's syndrome (TS) from the perspective of topological organization and functional connectivity within the DMN by electroencephalography (EEG) in resting-state.

**Methods:**

The study was conducted by analyzing the EEG data of TS patients with graph theory approaches. Thirty children with TS and thirty healthy controls (HCs) were recruited, and all subjects underwent resting-state EEG data acquisition. Functional connectivity within the DMN was calculated, and network properties were measured.

**Results:**

A significantly lower connectivity in the neural activity of the TS patients in the β band was found between the bilateral posterior cingulate cortex/retrosplenial cortex (t = -3.02, *p* < 0.05). Compared to HCs, the TS patients’ local topological properties (degree centrality) in the left temporal lobe in the γ band were changed, while the global topological properties (global efficiency and local efficiency) in DMN exhibited no significant differences. It was also demonstrated that the degree centrality of the left temporal lobe in the γ band was positively related to the Yale Global Tic Severity Scale scores (*r* = 0.369, *p* = 0.045).

**Conclusions:**

The functional connectivity and topological properties of the DMN of TS patients were disrupted, and abnormal DMN topological property alterations might affect the severity of tic in TS patients. The abnormal topological properties of the DMN in TS patients may be due to abnormal functional connectivity alterations. The findings provide novel insight into the neural mechanism of TS patients.

**Supplementary Information:**

The online version contains supplementary material available at 10.1186/s12883-023-03421-1.

## Introduction

Tourette's syndrome (TS) is a childhood-onset neurodevelopmental movement disorder characterized by multiple motor and vocal tics lasting more than a year prior to the age of 18 years old [1]. TS, once considered a rare condition, is now considered relatively common, and the prevalence of TS in school-age children worldwide is 0.3 to 0.9% [2]. Men are 3–4:1 times more likely to suffer from TS than women [3]. TS patients often have coexisting conditions such as attention-deficit/hyperactivity disorder (ADHD), obsessive–compulsive disorder (OCD) or obsessive–compulsive behavior, sleep disorders, and depression disorders [4]. TS may even lead to persistent social problems (e.g., bullying or isolation) [5].

At present, the etiology of TS is unclear, and its pathogenesis may be related to various factors. The abnormal brain structure and function of TS patients are thought to be one of the biological causes [6–8]. Previous studies found that this impaired brain activity might impact behavior, leading to motor, cognitive [9], and social problems [10]. With the development of neuroscience research, many noteworthy clues have been revealed in recent decades. Neurobiological models of TS suggest that abnormal connectivity of the prefrontal cortical-striatal-thalamic-cortex circuit may play a vital role in the course of the disease [11]. Functional magnetic resonance imaging (fMRI) studies have also found abnormalities in brain function in TS patients. For example, a recent resting-state fMRI study found that, compared to controls, patients with TS exhibited increased connectivity between the temporal gyri, insula, and putamen and between the orbitofrontal cortex and cingulate cortex [12]. In addition, other resting-state fMRI studies have identified alterations in brain networks, including default mode network (DMN) and frontal-parietal network in TS patients [13, 14], and abnormal alterations in brain function in the resting-state that may related to the severity of tic.

The DMN is one of the most active elements during the resting-state [15]. An increasing number of researchers have used the DMN to study the neural mechanism of neuropsychological diseases due to its distinctive features in the resting-state [16]. DMN is predominantly detected when a person becomes more concentrated internally rather than externally or on their internal mental-state processes, such as self-referential processing, theory of mind, autobiographical memory retrieval, self-processing, and emotion regulation [17]. The DMN has been conceptualized as a distributed brain network composed of several brain regions, including the anterior cingulate cortex (ACC), posterior cingulate/retrosplenial cortex (PCC/Rsp), medial prefrontal cortex (mPFC), and temporoparietal junction (TPJ). These brain regions showed high neural activity and temporal synchrony in the resting-state [15].

Neurological alterations within the DMN have been found in a variety of psychiatric disorders, such as ADHD [18], autism [19], and major depression [20]. For TS, a study based on fMRI found functional connectivity disruptions in the inner DMN region [21]. Additionally, functional connectivity within the DMN correlated negatively with tic severity [13]. Further, Openneer et al. [14] showed lower local efficiency and clustering coefficient values in DNM of TS patients, specifically for TS without comorbid ADHD, compared to healthy controls (HCs). They also observed a negative association between tic severity and local efficiency and clustering coefficient in the DNM. Moreover, a previous study has found abnormal brain regions involved in DMN of TS patients [22]. However, these literatures investigated the neural activity during the tic suppression, and the findings reflected an effect of tic suppression rather than a true resting-state. Therefore, the main aim of the present study was to investigate the alterations in DMN in a true resting-state.

Electroencephalography (EEG) is a low-cost, noninvasive method of measuring brain activity. As a simple millisecond-resolution readout of brain activity, this technique, when combined with standardized analytical techniques, can be used not only to understand the physiological function of subjects but also to reflect pathological alterations. Although several theories have been proposed about the causes of TS, little is known about how TS is explained at the neural level. One way to solve this problem is to study how the brain activity in patients with TS's DMN nodes communicates via EEG during the resting-state.

Previous studies have identified abnormalities in functional brain activity and interactions between some brain regions in TS patients using EEG. Although these findings have shed some light on the abnormal central mechanisms of TS, they do not provide information on large-scale neuronal communication in the human brain because they are based on brain activation patterns to find abnormal activities in individual brain regions rather than on the central mechanisms of the disease at the systemic level. Suppose the human brain is studied as an integrated network of functionally interacting brain regions. In that case, it can further reflect alterations in the brain's communicative activity as a complex system at different spatial scales by exploring how its functional connectivity and information integration interrelate with human behavioral activities.

Given the diversity and complexity of brain networks, graph theory, as a data-driven technique, is particularly well suited for comprehensive studies that reveal inherent functional connectivity patterns and complex brain network features [23, 24]. A recent study used graph theoretical analysis to examine brain networks in TS and found that topological properties might serve as a reliable biomarker to differentiate TS patients from healthy controls [25]. This suggests that graph theory analysis can provide researchers with a good understanding of the neural basis of TS. Therefore, the main objective of our study was to investigate whether DMN connectivity was altered in TS patients. Subsequently, graph theory-based analysis was used to investigate whether the DMN topological properties of TS patients had abnormal alterations. We hypothesized that in patients with TS, the DMN functional connectivity and topology had altered, and these alterations might be associated with the severity of TS. According to previous studies, we used two methods (lagged phase synchronization [LPS] and graph theory) for assessing the DMN [26, 27].

## Materials and methods

### Participants

Thirty children with TS were recruited from West China Second University Hospital. Eligible participants were identified using the Diagnostic and Statistical Manual of Mental Disorders, Fifth Edition (DSM-5). Additional inclusion criteria were: (1) normal vision and hearing; (2) Han Chinese ethnicity. The exclusion criteria included (1) one or more comorbid mental disorders (e.g., ADHD, OCD, intellectual disability, learning disability, or conduct disorder); (2) severe physical disease or brain injury; (3) past or present use of any psychotropic substance, including stimulants and other drugs; and (4) inability to cooperate with EEG acquisition for any reason. Meanwhile, thirty age and biological sex-matched HCs were included in the study as a control group. Written consent was obtained from participants and their parents. The protocol for the study was carefully reviewed, accepted, and approved by the local Medical Research Ethics Committee of West China Second University Hospital, Sichuan University.

### Clinical assessment

The severity of tics was assessed by the Yale Global Tic Severity Scale (YGTSS) [28]. This clinician-rated, semi-structured interview provides a quantitative measure of tic severity. The YGTSS rates movement and vocal tics on dimensions such as number, frequency, intensity, complexity, and interference. Each dimension is scored on a five-point scale. The total severity scores are obtained by summing all scores across vocal and motor tics. It also includes a separate impairment rating. The sum of the total tics and impairment scores determines the global severity scores (YGTSS scores).

### EEG Acquisition

The EEG recordings were made using 19 Ag/AgCl electrodes placed on the scalp at Fp1, Fp2, Fz, F3, F4, F7, F8, Cz, C3, C4, Pz, P3, P4, T3, T4, T5, T6, O1, and O2 electrodes sites, following the 10/20 international electrode placement [29]. EEG data were sampled at 500 Hz and bandpass filtering of 0.3–70 Hz. Electrode impedance was always kept below 5 kΩ [30]. Subjects were asked to sit on chairs in a quiet room, close their eyes, and stay awake. During this period, EEG datasets were collected for 15 min. Subjects were not asked to suppress their tics while EEG was recorded. Meanwhile, a trained EEG technician performs quality control over EEG acquisition in the next room.

If the technicians noted a significant deviation from the study protocol (e.g., non-tic-induced finger tapping, looking from side to side, eyebrow raising, etc.), the task was paused and the participant was provided verbal feedback regarding adherence to the instructions. In cases where the participant failed to follow instructions, data acquisition was stopped, and the participant was re-instructed to perform the task before resuming data acquisition. If the participant required multiple re-instructions, data acquisition was suspended, and the participant was excluded from further data analysis.

### EEG Preprocessing

The EEGLAB toolbox [31] (available at sccn.ucsd.edu/eeglab) based on MATLAB was used to preprocess the collected EEG data. First, an expert EEG technician reviewed the data and excluded blink, muscle, and electrocardiograph artifacts by visual inspection. In addition, EEG data with artifacts due to movements during tic expression were also excluded from collection. The lengths of EEG epochs free of artifacts were varied from 36 to 92 s. Then, the remaining preprocessing steps included averaging referencing, 0.5–45 Hz bandpass filtering [32], 4 s data segmentation [33, 34], and artifact removal by independent component analysis decomposition [35].

### EEG Source Localization

To search for the active sources of the scalp potentials, Exact Low Resolution Electromagnetic Tomography (eLORETA) (http://www.uzh.ch/keyinst/loreta.htm) was used to perform a source localization analysis of all frequency oscillations in the resting-state. The LORETA mechanism is a discrete, three-dimensional distributed, linear, weighted minimum norm inverse solution and has the ability to reconstruct intercortical activity with correct localization from scalp EEG data [36, 37]. Moreover, LORETA has no localization bias, even in the existence of noise. Most of all, although clinical EEG assessments typically use 19 scalp electrodes, the LORETA software benefits from its excellent localization agreement and is therefore also considered suitable for studying DMN when using the standard 19-electrode EEG [38]. For the present study, 19 electrode coordinates were first created. Then, an average head model was interpolated on this basis, which was necessary to calculate the "conversion matrix" for the conversion of the electrical potential differences recorded at the scalp level into "current density." The pre-processed EEG is converted and imported into LORETA to create an "EEG cross-spectrum." Corresponding functional images of the cortical distribution of different frequency bands of the generators of oscillatory electrical activity were then calculated and elaborated. According to the difference in the frequency, the following frequency bands were defined: δ (0.5–4 Hz); θ (4–8 Hz); α (8–13 Hz); β (13–30 Hz); and γ (30–45 Hz) [39].

### Functional connectivity analysis

Seed-based functional connectivity has been widely used [40]. To evaluate the connectivity in the DMN, according to a previous study [41], 12 regions of interest (ROIs) were defined, as shown in Supplement Table 1. The intercortical surfaces were parcellated into 15,000 anatomical vertices based on Montreal Neurological Institute templates [42]. Because the single centroid voxel (the closest to the center of the ROI) is an excellent representative of the corresponding ROI, for the analysis of connectivity between ROIs, a method using a single voxel at the centroid of each ROI was chosen.

**Table 1:** Demographic characteristics

	TS	HC	*p*
Age (year)	7.08 ± 2.36	7.73 ± 3.15	0.37
Male (%)	22(73.33%)	22(73.33%)	1
YGTSS scores	32.23 ± 13.29		

The method of connectivity analysis was based on the LPS. The LPS quantifies the nonlinear relationship between ROIs after excluding the instantaneous zero-lag contribution. Zero-lag synchronization is usually caused by non-physiological artifacts such as volume conduction and low spatial resolution, which usually affect other connection indices[43]. Therefore, this correction is important.

### Network measures

Based on the network constructed above, we analyzed its characteristics. For this purpose, we calculated the average connectivity matrix for all subjects. To compare with previous literature about TS [14, 44], four metrics were chosen to measure the network properties of all subjects (2 global topology parameters and 2 node topology parameters) using the GRETNA toolbox (https://www.nitrc.org/projects/gretna). An extended description of the topological parameters of the brain network used in this study can be found in this article [24].

Global efficiency: global efficiency measures the global efficiency of parallel information transfer in a network. The shorter the shortest path length, the higher the global efficiency of the network, and the faster the information transfer rate between network nodes.

Local efficiency: the local efficiency of the network measures how efficient communication is among the first neighbors of a given node when it is removed.

Clustering coefficient: the clustering coefficient of a given node measures the likelihood of its neighbors being connected to each other, which is equal to the ratio of the number of edges actually connected between the neighbors of that node to the maximum number of possible connected edges.

Degree centrality: the nodal degree for a given node reflects its information communication ability in the functional network. Degree centrality demonstrates the total strength of direct functional connections between local brain regions and the whole brain. Regions with high degree centrality values often represent the core nodes of brain networks to each other.

### Statistics

To analyze potential between-group differences in demography, two-sample t-tests were conducted. Tests were performed to compare the LPS values and topological properties (global efficiency, local efficiency, clustering coefficient, and degree centrality) between TS patients and HCs in every frequency band. EEG functional connectivity data were compared using the statistical nonparametric mapping (SnPM) method based on Fisher's permutation [45] provided by the eLORETA software. This method is based on Fisher's permutation test: a subset of nonparametric statistics. Specifically, using this approach, significant differences (*p* < 0.05) were identified by comparing the distribution of permutated values at the voxel level using a nonparametric permutation procedure [46]. Correction for multiple comparisons in SnPM with random permutations (5000 in the current study) has been shown to yield results similar to those obtained from statistical parametric mapping with a general linear model with multiple comparison corrections derived from random field theory [46]. Since comparing networks consisting of different numbers of edges may lead to pseudo-differences due to differences in network topology [47], we binarized the connectivity matrix using different thresholds to compare graphs with a fixed network density. The integrated area under the curve (AUC) is very sensitive to alterations in the topological properties of the brain network [48]. Therefore, the AUC was used to identify the significant between-group differences in the topological properties of the DMN between the TS and HC groups. We calculated the AUC of each topological property metric over the range of 0.05 to 0.50 with an interval of 0.01. Bonferroni-corrected two-sample t-tests were conducted to analyze the differences in topological properties.

Moreover, Pearson correlations were computed between the YGTSS scores and brain activity to investigate the relationship between the YGTSS scores and these EEG metrics with significant between-group differences. *p* < 0.05 was considered statistically significant.

## Results

### Demographics

As shown in Table 1, TS patients (22 males and 8 females) had a mean age of 7.08 years (standard deviation [SD]: ± 2.36) and a YGTSS scores of 32.23 (SD: ± 14.81). The HCs (22 males and 8 females) had a mean age of 7.73 years (SD: ± 3.15).

### Functional Connectivity

**Fig. 1:**
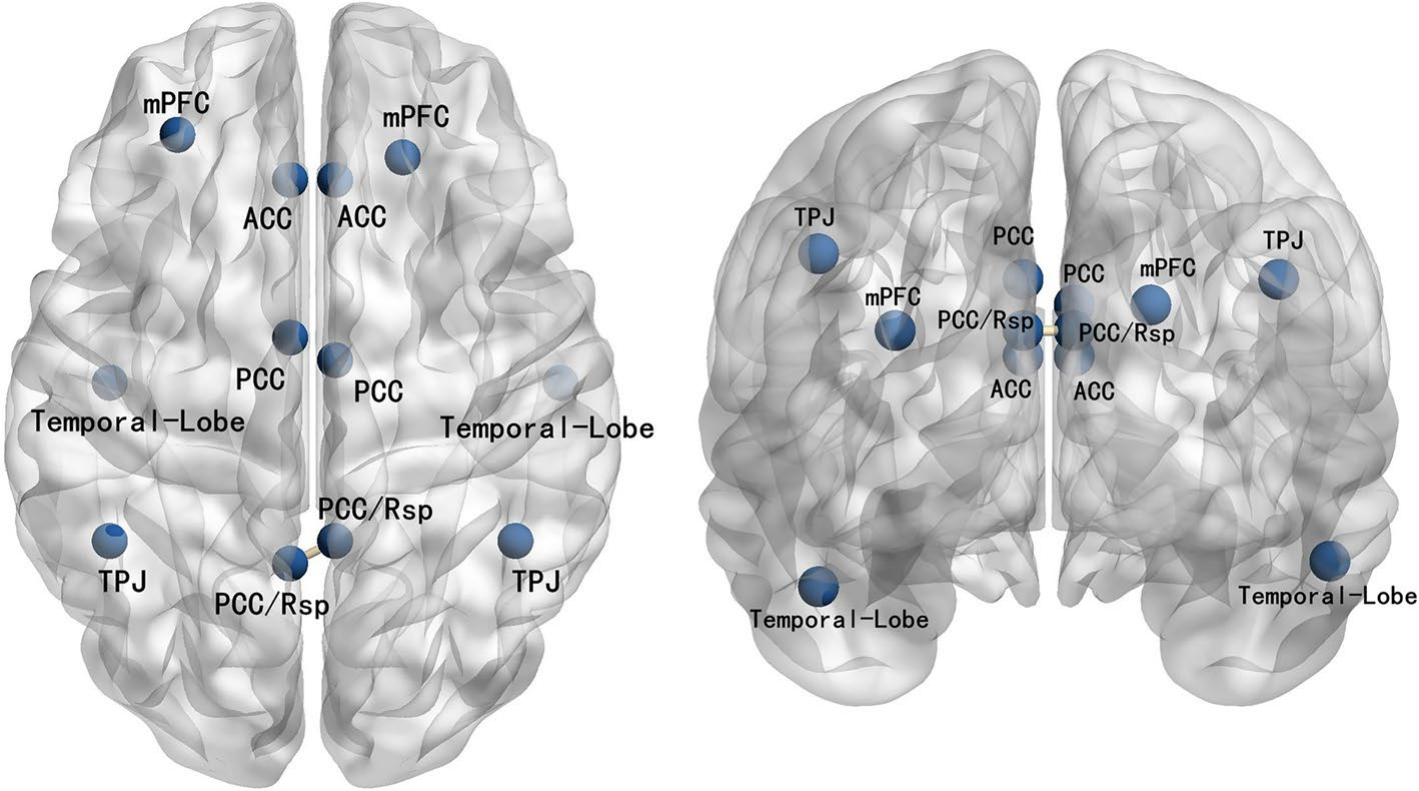
Abnormal DMN interregional functional connectivity between patients with TS and HCs

The functional connectivity analyses were performed based on LPS. There were statistically significant alterations in LPS values in patients with TS compared to HCs for β band activity in the DMN. More specifically, greater connectivity was found between the bilateral PCC/Rsp across both hemispheres in the β band of the TS patients (t = 3.581, *p* < 0.05). (Fig. 1).

### Topological Properties

The global efficiency and local efficiency were used to measure the global properties. However, the global and local efficiency showed no significant difference between the two groups in the five frequency bands (Supplementary Fig. 1).

Two network parameters, nodal clustering coefficients and nodal degree centrality, were used to measure the nodal properties of the functional brain network in both groups of subjects. Compared to the HC group, TS patients showed increased clustering coefficient values in the δ band in the left ACC and the right mPFC, while decreased in the right temporal lobe and the rightt TPJ. In addition, TS patients showed decreased clustering coefficient value in the left PCC in the β band and increased clustering coefficient value in the left PCC in the γ band. However, after correction for multiple comparisons, these differences in clustering coefficients were non-significant (see Supplementary Table 2 for the statistical values). As for the degree centrality, TS patients showed increased value in the left temporal lobe in the γ band (t = 2.845, *p* = 0.004), and the difference still existed after correction for multiple comparisons.

### Association between YGTSS and EEG Metrics

**Fig. 2:**
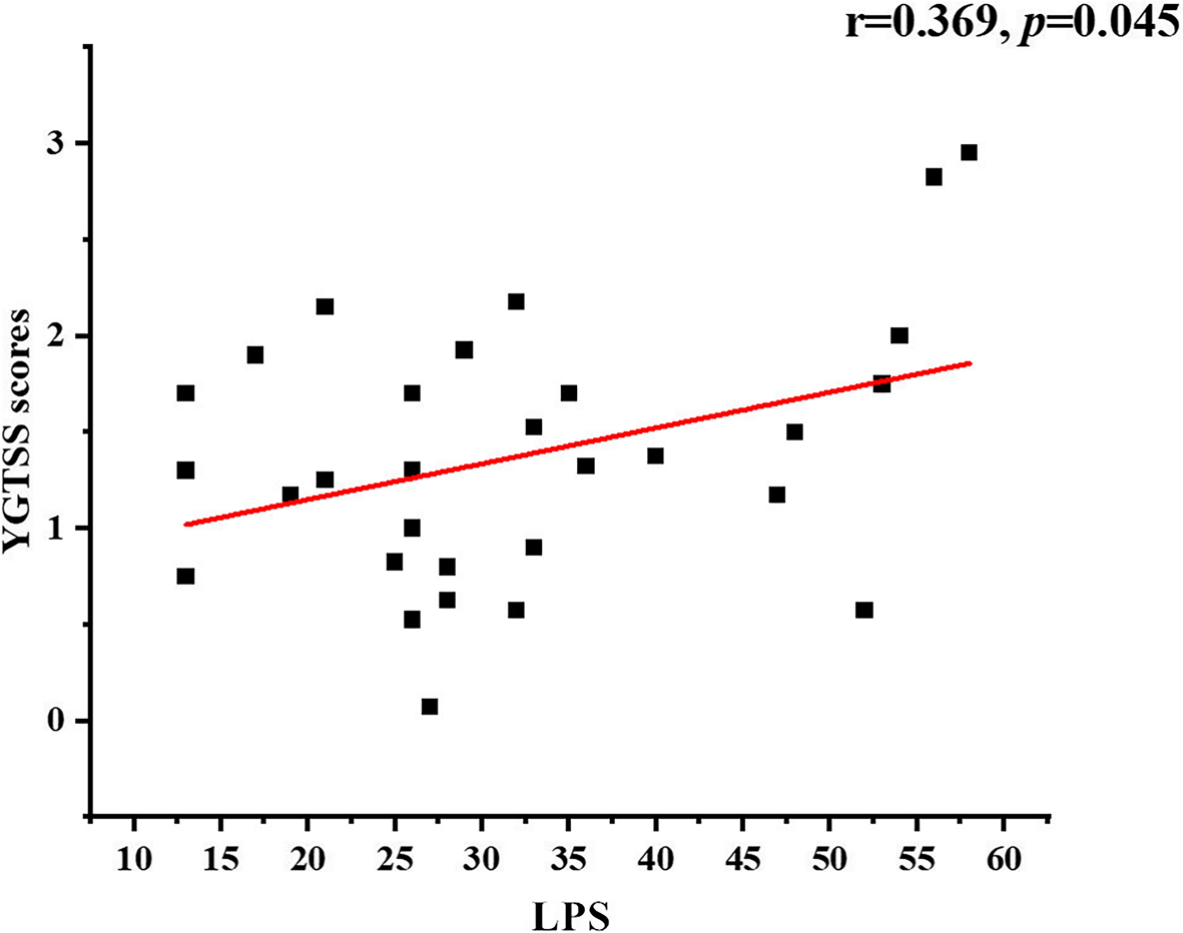
In TS patients, the LPS in the right temporal lobe in the γ band showed a positive correlation with YGTSS scores

Pearson correlation was used to evaluate the linear correlation between the YGTSS scores and these EEG metrics with significant between-group differences. Only the degree centrality was associated with total YGTSS scores. Specifically, YGTSS scores were positively related to the degree centrality in the left temporal lobe in the γ band (r = 0.369, *p* = 0.045, Fig. 2).

## Discussion

This cross-sectional study aimed to elucidate further functional network alterations in children with TS at the source level using brain network analysis utilizing EEG techniques. A strong increase in the neural connectivity of the TS patients in the β band was found between the bilateral PCC/Rsp. Furthermore, the topological properties of the DMN have altered. Specifically, the local topological properties in DMN of TS patients were altered (TS patients showed increased degree centrality value in the left temporal lobe in the γ band). In contrast, the global topological properties (global and local efficiency) exhibited no significant differences. In addition, the degree centrality of the left temporal lobe was positively correlated with YGTSS scores.

Consistent with our expectations and a prior neuroimaging report[13], our findings revealed greater connectivity within the DMN in TS patients. The DMN is thought to be involved in processes related to self-awareness, such as self-reference and autobiographic memory retrieval[49]. The result indicated that a network known to underlie self-referential processing might also contribute to the neural mechanism of TS. Increased connectivity within the DMN may thus represent a neural correlate of self-referential thinking related to tics, e.g., in the context of the premonitory urge feeling [50]. In addition, the increased connectivity was found between the bilateral PCC/Rsp. The PCC/Rsp was an important hub in DMN and plays a vital role in cognitive control [51, 52]. Previous studies concluded that TS patients accomplish various cognitive tasks through enhanced cognitive control because of their chronic need to cope with physically generated unwanted behaviors, specifically motor and vocal tics[53, 54]. Therefore, we speculated that the increased bilateral PCC/Rsp functional connectivity in TS patients found in the present study might be related to a compensatory cognitive mechanism that develop in TS patients due to long-term tic suppression. However, since this is a cross-sectional study, and the compensatory process cannot be directly tested, this speculation has yet to be confirmed.

Moreover, this result was found in the β band. Consistent with our findings, Zapparoli et al. [55] also found that the abnormal modulation of the EEG rhythm in TS was specific for the β frequency. In fact, it has been hypothesized that β oscillations may represent a specific tendency of the sensorimotor system to maintain the "status quo" and represent the "idle rhythm" of the motor system. And the β oscillations and/or coupling in the β band are expressed more strongly if the maintenance of the status quo is intended or predicted than if a change is expected [56]. This hypothesis is strongly supported by studies related to movement disorders (e.g., Parkinson's disease) in which people with Parkinson's disease have difficulty initiating or changing their movements, which is significantly associated with higher levels of β oscillations [57]. Although there was no evidence for this, we speculated that an increase in β bands was observed because TS patients try to inhibit involuntary movements for long periods in their daily lives.

This study also investigated the nodal topological characteristics of functional network alterations in TS patients. Degree centrality measures the number of instantaneous functional connections (or correlations) between a given voxel (node) and the rest of the brain, rather than with specific nodes or networks [58]. Thus, this metric allows us to quantify the importance of a node to the rest of the brain. The brain network considers nodes with a high degree centrality “hubs”. In this study, we found that TS patients in the γ band showed increased degree centrality values in the left temporal lobe, meaning that the left temporal lobe was hyperactive in the γ band. The temporal lobe has previously been implicated in TS. A previous study also found a thinner cortex of the temporal lobes of TS subjects [59], and the author interpreted it as a cortical folding abnormality. The temporal lobe is part of the limbic system, i.e., amygdalae and hippocampus. A previous study found that TS patients showed stronger activity within the amygdala/hippocampus complex during spontaneous than voluntary tics, suggesting that activity in these regions may represent features of the premonitory urges that generate spontaneous tic behaviors [60]. Combined with our findings, this further suggested that the temporal lobe might be an important brain region associated with the etiology of TS.

This study also found a correlation between tics severity and EEG metrics with significant intergroup differences in patients with TS. Specifically, YGTSS scores were positively correlated with the value of degree centrality in the left temporal lobe in the γ band. This study's results further confirm the temporal lobe's role in TS. The literature suggested that the temporal lobe was associated with the direct control of urge inhibition [61]. As mentioned previously, we speculated that TS patients need to continuously and consciously suppress involuntary body tics in their daily life, resulting in enhanced temporal lobe function and increased connectivity with other brain regions. The more severe the tics symptoms, the more the temporal lobes are activated and the stronger the connections with other brain regions. In future research, examining correlations between the brain activity reported here and premonitory urge scores will directly assess this hypothesis. Previous studies found that insula [62], supplementary motor area [63], and regions of the cingulate [64] were most commonly implicated in premonitory urge. Therefore, the temporal lobe may play a “hub” role in communicating with these brain regions. However, to date, it is not clear whether the temporal lobe is involved in the pathogenesis of TS. If so, the findings would help improve targeted therapy in the future. For example, some treatments used to improve local brain function can be used in TS patients, such as transcranial magnetic stimulation, deep brain stimulation, and transcranial direct current stimulation. These therapies can provide compensatory improvement to localized functionally enhanced or weakened brain areas and promote localized brain area functional recovery [65].

The clustering coefficient is equivalent to the fraction of nodal neighbors that are also neighbors to each other [66]. Thus, the higher clustering coefficient indicates high local efficiency, more stability, and increased functional segregation in the disrupted brain regions. In this study, we found that compared to the HCs, the clustering coefficients of the right mPFC, the left ACC, the right temporal lobe, the left PCC, and the right TPJ demonstrated a trend of change in certain bands. These nodes, especially the mPFC, are essential nodes within the DMN and play central roles in the neuropathology of TS. Similarly, studies based on the brain structure of TS patients also found the prefrontal area to be involved in the onset of tics [67], and the increase in prefrontal cortical thickness was correlated positively with tic severity [59]. Brain networks are thought to evolve to maximize the cost efficiency of parallel information processing (i.e., high efficiency of parallel information transfer at low costs) [68]. Although not yet confirmed, we speculated that the symptoms associated with TS disease may place abnormal demands on brain function to work as efficiently as possible. This may lead to alterations in the topological properties of specific networks.

The strength of this study is that the included subjects with TS did not have other comorbid neuropsychological disorders, such as ADHD or OCD. In addition, none of the subjects included in the study had any treatment before this research, including pharmacological and psychologically assisted treatment. This ensured that the sample for this study was homogeneous, and the selection of a homogeneous group ensured that potential confounders minimized the impact of the results. Although selecting a homogenous group assured minimal influence of potential confounders on results, our findings may not be generalizable to children with comorbid disorders such as OCD or ADHD, both of which are very common in patients with TS [69]. Therefore, future studies using similar techniques should be carried out in TS patients with comorbidities and also OCD and ADHD patients without TS for comparison.

There are some other limitations must be acknowledged in our study. First, gender and handedness may influence the results; however, due to the relatively small sample size, it was impossible to standardize gender and handedness. Second, although EEG may have some advantages over fMRI, such as direct measurement of neural oscillations, better temporal resolution, and high feasibility of use in TS patients, a known limitation of EEG is its reduced spatial accuracy. Finally, a relatively simple methodological approach was adopted. This study adopted a semirealistic head model with individual electrodes and sensor locations rather than a realistic head model. Therefore, in future studies, both EEG and other brain imaging techniques (for example, MRI, X-ray, and CT) should be performed on the subjects, combining these two examination methods to improve the study's precision [70].

## Conclusion

To sum up, we used a graph theory approach to investigate the topological reorganization of the DMN in TS patients. Subjects with TS exhibited abnormal functional connectivity and topological properties (nodal topology parameters) within DMN in specific frequency bands. In addition, abnormal functional connectivity and topological properties were associated with the severity of tics in patients with TS. Therefore, we assumed that abnormal functional connectivity and topological properties might be potential biomarkers for objective diagnoses of TS. Furthermore, the findings gave a novel insight into the neural mechanism of TS patients.

### Supplementary Information

Below is the link to the electronic supplementary material.

Supplementary file1 (DOCX 104 kb)

## Data Availability

The data that support the findings of this study are available on request from the corresponding author. The data are not publicly available due to privacy or ethical restrictions.

## Abbreviations

ACC: Anterior cingulate cortex
AUC: Area under the curve
ADHD: Attention-deficit/hyperactivity disorder
DMN: Default mode network
EEG: Electroencephalography
eLORETA: Exact Low Resolution Electromagnetic Tomography
fMRI: Functional magnetic resonance imaging
HCs: Healthy controls
LPS: Lagged phase synchronization
mPFC: Medial prefrontal cortex
OCD: Obsessive–compulsive disorder
PCC/Rsp: Posterior cingulate/retrosplenial cortex
ROI: Region of interest
SD: Standard deviation
SnPM: Statistical nonparametric mapping
TPJ: Temporoparietal junction
TS: Tourette's syndrome
YGTSS: Yale Global Tic Severity Scale
