# Supplementary material for: Alterations in the topological organization of the default-mode network in Tourette syndrome
Source: BMC Neurol. 2023 Oct 30;23:390. doi: 10.1186/s12883-023-03421-1 (PMC10614376; doi:10.1186/s12883-023-03421-1)
Supplement: Supplementary file 1 — Supplementary file1 (DOCX 104 kb) [file 12883_2023_3421_MOESM1_ESM.docx]

Supplementary Table 1. Twelve cortical regions of interest.

| ROIs | MNI coordinates | | | Anatomical regions | Brodmann areas |
| --- | --- | --- | --- | --- | --- |
|  |  | | |  |  |
|  | x | y | z |  |  |
| 1 | -30 | 40 | 25 | Left mPFC | 8-9-10 |
| 2 | 20 | 35 | 30 | Right mPFC | 8-9-10 |
| 3 | -45 | -15 | -25 | Left Temporal Lobe | 21-28-36 |
| 4 | 55 | -15 | -20 | Right Temporal Lobe | 21-28-36 |
| 5 | -5 | -5 | 35 | Left PCC | 23-24 |
| 6 | 5 | -10 | 30 | Right PCC | 23-24 |
| 7 | -5 | 30 | 20 | Left ACC | 32 |
| 8 | 5 | 30 | 20 | Right ACC | 32 |
| 9 | -5 | -55 | 25 | Left PCC/Rsp | 29-30-31 |
| 10 | 5 | -50 | 25 | Right PCC/Rsp | 29-30-31 |
| 11 | -45 | -50 | 40 | Left TPJ | 39-40 |
| 12 | 45 | -50 | 35 | Right TPJ | 39-40 |

Abbreviation: medial prefrontal cortex, mPFC; posterior cingulate cortex, PCC; anterior cingulate cortex, ACC; posterior cingulate/retrosplenial cortex, PCC/Rsp; temporoparietal junction, TPJ.

Supplementary Table 2.Statistical values of clustering coefficients.

| Band | Region | t | *p* |
| --- | --- | --- | --- |
| δ | right mPFC | 2.1 | 0.04 |
|  | left ACC | 2.193 | 0.033 |
|  | right temporal lobe | -2.268 | 0.027 |
|  | right TPJ | -2.769 | 0.008 |
| β | left PCC | -2.624 | 0.011 |
| γ | left PCC | 2.09 | 0.04 |
| Abbreviation: medial prefrontal cortex, mPFC; anterior cingulate cortex, ACC; posterior cingulate cortex, PCC; temporoparietal junction, TPJ. | | | |


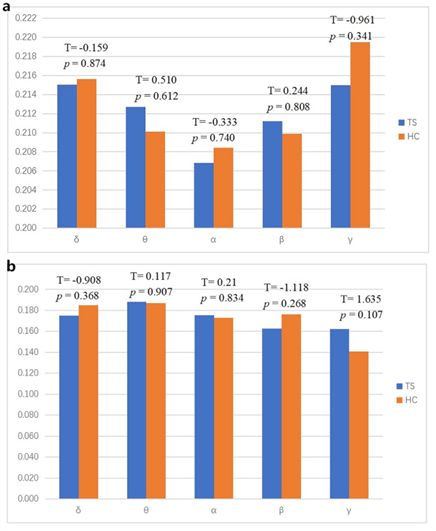


Supplementary Fig. 1. The global efficiency (a) and the local efficiency (b) of the DMN between patients with TS and HCs.
